# Special Issue: “Recent Developments in Geopolymers and Alkali-Activated Materials”

**DOI:** 10.3390/ma17010245

**Published:** 2024-01-02

**Authors:** Sujeong Lee

**Affiliations:** 1 Resources Utilization Division, Korea Institute of Geoscience and Mineral Resources, 124 Gwahang-no, Yuseong-gu, Daejeon 34132, Republic of Korea; crystal2@kigam.re.kr; 2 Resources Engineering Department, Korea National University of Science and Technology, 217 Gajeong-ro, Yuseong-gu, Daejeon 34113, Republic of Korea

As efforts toward global sustainability converge with the imperative to reduce the environmental impact of construction materials, extensive research and development is underway in the field of geopolymers and alkali-activated materials (AAMs). This Special Issue aims to comprehensively present the latest research findings, methodologies, and crucial insights from leading researchers and practitioners in this field.

Our contributors, comprising outstanding researchers, scholars, and industrial experts, have each brought forth new perspectives to enhance our collective understanding of geopolymers and AAMs. Geopolymers and AAMs are gaining significant attention not only for their potential to replace traditional materials but also for surpassing performance expectations and serving as low-carbon, environmentally friendly technologies to address climate change. The unique properties of geopolymers, such as their high strength, durability, and fire resistance, reinforce their position as eco-friendly alternatives. Moreover, AAMs broaden the horizon of sustainable construction materials by incorporating a wide range of raw materials, such as industrial by-products, waste, and biomass ash. This not only makes a significant contribution to environmental sustainability but also enhances the durability and overall performance of AAMs.

While past research primarily focused on understanding and improving the chemical and physical properties of geopolymers and AAMs, recent studies actively explore the utilization of various industrial by-products, waste materials, and biological sources as raw materials. Research efforts are also directed toward predicting material properties using machine learning, optimizing mix designs, and forecasting the characteristics of geopolymers and AAMs. These materials find applications not only in construction but also in diverse fields such as nanomaterials, aerospace, ceramics, and space exploration. Despite these advancements, there is an ongoing need for fundamental research, standardization, and specification improvements for geopolymers and AAMs.

This Special Issue features eight research papers and two review papers covering a spectrum of topics. Ricciotti et al.’s [1] study demonstrates the production of metakaolin geopolymer-based plaster by recycling waste generated in the production process of porcelain stoneware products, with excellent adhesion suitable for the restoration and preservation of artworks. Zue et al. [2] showcase the synthesis of low-temperature C-S-H using the alkali activation of high-carbon biomass fly ash, a challenging material to recycle. Amin et al. [3] employ machine learning to predict the mechanical properties of 156 geopolymer concrete samples, exploring the efficiency of machine learning in enhancing the production process of geopolymers. Kim et al. [4] investigate the positive influence of Ca additives on the early strength of geopolymers, providing advanced insights into phase evolution through a Rietveld refinement analysis. Ibraheem et al. [5] explore the diversification of raw materials in AAMs by adding quarry rock dust and steel fiber, presenting effective ways to recycle waste resources and enhance strength. Kim et al. [6] manufacture a porous alkali-activated material using zinc powder as a foaming agent, introducing a novel reaction pathway compared to commonly used aluminum powder. Rozek et al. [7] report on the performance of boroaluminaosilicate geopolymers in immobilizing heavy metals, surpassing traditional cement concrete in metal fixation capabilities. Terrones-Saeta et al. [8] demonstrate the production of ceramics with sufficient strength using chamotte as an alumino-silicate raw material and potassium-rich biomass bottom ash as an alkali activator. Ahmed et al. [9] provide a specialized analysis of geopolymer recycled aggregate concrete made from recycled aggregates and geopolymers in terms of structural applications. Lee and Riessen [10] analyze the feasibility of manufacturing geopolymers from lunar-simulant-based materials originating from lunar regolith, highlighting the growing interest in lunar exploration. 

This Special Issue highlights the synergy between academia, industry, and research institutions, confirming their pivotal role in the continuous advancement of this field. The collaborative efforts showcased on these pages signify the scientific community’s dedication to adapting to ongoing global changes.

We extend our sincere gratitude to all the authors who contributed to this Special Issue. Their invaluable research contributions made this Special Issue possible, and we genuinely appreciate their efforts in advancing the sustainable development of construction materials.

In conclusion, we hope that this Special Issue serves as a comprehensive resource, fostering innovation and dialogue among researchers, engineers, policymakers, and industry experts in this dynamic field.
